# Improving Functional Capacity and Quality of Life in Parkinson’s Disease Patients through REAC Neuromodulation Treatments for Mood and Behavioral Disorders

**DOI:** 10.3390/jpm13060937

**Published:** 2023-06-01

**Authors:** Chiara Rinaldi, Cleuton Braga Landre, Maria Izabel Volpe, Rebeca Góes Gonçalves, Lucas dos Santos Nunes, Danyela Darienso, Ana Vitoria Cruz, João Douglas Oliveira, Salvatore Rinaldi, Vania Fontani, Ana Rita Barcessat

**Affiliations:** 1 Department of Neuroscience, Psychology, Drug Area, and Child Health (NEUROFARBA), University of Florence, 50121 Florence, Italy; 2 Department of Regenerative Medicine, Rinaldi Fontani Institute, 50144 Florence, Italy; srinaldi@irf.it (S.R.); vfontani@irf.it (V.F.); 3 Department of Adaptive Neuro Psycho Physio Pathology and Neuro Psycho Physical Optimization, Rinaldi Fontani Institute, 50144 Florence, Italy; 4 Department of Biological and Health Sciences, Federal University of Amapá—UNIFAP, Macapá 68903-419, Brazilrebecagoes018@gmail.com (R.G.G.); nlucas499@gmail.com (L.d.S.N.);; 5 Graduate Program in Health Sciences—PPGCS, Federal University of Amapá—UNIFAP, Macapá 68903-419, Brazil; 6 Department of Biomedical Sciences, University of Sassari, 07100 Sassari, Italy

**Keywords:** Parkinson, motor symptoms, non-motor symptoms, mood and behavioral disorders, neuromodulation, radio electric asymmetric conveyer technology

## Abstract

Parkinson’s disease is a neurological disorder that affects both motor and non-motor functions, including depression, anxiety, and cognitive decline. Currently, it remains a challenge to distinguish the correlation between these aspects and their impact on one another. To try to clarify these reciprocal influences, in this study we have used specific radio electric asymmetric conveyer (REAC) technology neuromodulation treatments for behavioral mood disorders and adjustment disorders. In particular, we employed the neuro-postural optimization (NPO) and neuro-psycho-physical optimization (NPPOs) treatments. The study enrolled randomly 50 subjects of both genders previously diagnosed with Parkinson’s disease for at least six months. Prior to and following REAC NPO and NPPOs treatments, we assessed the subjects using functional dysmetria (FD) evaluation, five times sit to stand test (FTSST) for postural stability, and the 12-item Short-Form Health Survey (SF-12) for quality of life (QLF) evaluation. The positive results produced by the REAC NPO and NPPOs neuromodulation treatments, specific for mood and adaptation disorders, on dysfunctional motor disorders, and quality of life confirm how the non-motor components can condition the symptomatology of Parkinsonian motor symptoms. These results also highlight the usefulness of REAC NPO and NPPOs treatments in improving the overall quality of life of these patients.

## 1. Introduction

Parkinson’s disease (PD) is a debilitating neurodegenerative disorder that affects millions of people worldwide. It is characterized by the loss of dopamine-producing neurons in the brain, which leads to both motor and non-motor symptoms [1,2,3]. Motor symptoms include tremors, rigidity, bradykinesia, and postural instability, while non-motor symptoms include depression, anxiety, cognitive dysfunction [4,5], adjustment disorder (AD), and stress-related disorders.

Recent research has suggested that environmental exposures [6,7] throughout an individual’s life, known as the exposome [8], may contribute to the development and progression of PD through epigenetic modifications [9] that affect genes encoding for dopamine. These modifications can alter the expression and function of proteins involved in dopamine synthesis, storage, release, and signaling, leading to changes in dopamine levels in the brain and the motor and non-motor symptoms associated with PD, such as depression, anxiety, and adjustment disorders.

Depression is the most frequently observed psychiatric disorder in PD [10,11] and is associated with worsening of motor function [12] and a more severe clinical picture of PD. Studies suggest that depression in PD may result from both neuropathology and psychological and physical limitations of the patient. Anxiety symptoms may also be observed in PD patients, with varying incidence rates, ranging from 3.6% to 40%.

Understanding the correlations between motor and non-motor symptoms in PD is crucial for effective management of the condition [13]. Recent research has shed light on the potential correlations between adjustment disorder (AD) and the onset and exacerbation of Parkinsonian symptoms [14,15]. AD is one of the most commonly diagnosed mental health disorders and is generally conceptualized to be mild and short-lived. Despite the frequent use of AD in clinical settings, little is known about the prognosis of this condition [16].

Parkinson’s disease patients may exhibit emotional and behavioral symptoms similar to those of AD, including anxiety, depression, irritability, and withdrawal, as they navigate the challenges of their condition, such as mobility problems, tremors, and speech or swallowing difficulties. To investigate these correlations and reciprocal influences between motor and non-motor symptoms in PD, this study employed Neuro-Postural Optimization (NPO) and Neuro-Psycho-Physical Optimization (NPPOs) treatments of Radio Electric Asymmetric Conveyer (REAC) technology. These treatments have been studied to improve neuropsychic and physical performance in the broad field of mood, behavior, and adaptive disorders through a positive remodulation of neuronal endogenous bioelectrical activity (EBA) [17].

In this paper, we will discuss the potential correlations between environmental exposures, epigenetic modifications, and the onset and progression of PD symptoms, with a particular focus on the non-motor symptoms of depression, anxiety, and adjustment disorder. We will also present the results of our study, which used NPO and NPPOs treatments of REAC technology to investigate the reciprocal influences between motor and non-motor symptoms in PD patients. Our findings have the potential to improve the understanding and management of PD, particularly in terms of the non-motor symptoms that have a significant impact on patients’ quality of life.

## 2. Materials and Methods

### 2.1. Study Design

This single-arm trial was conducted at the Health Department’s Laboratory at the Federal University of Amapá (UNIFAP) in Macapá, Brazil. The study was approved by the UNIFAP Ethics Committee in accordance with its opinion 5.232.551.

### 2.2. Power Analysis

We calculated the statistical sample size using GPower [18,19] software version 3.1.9.4. Using the following parameters: effect size of 0.50, α probability error of 0.05, power of 0.95, and the Wilcoxon signed-rank test. Based on these parameters, the sample size calculation yielded a sample size of 34 subjects. To mitigate potential dropouts by some participants, we increased the number of patients in the study.

### 2.3. Inclusion and Exclusion Criteria

The study included adult male and female participants who had received a clinical diagnosis of Parkinson’s disease at least six months prior to enrollment and demonstrated the willingness and ability to understand and comply with study requirements.

Participants with cognitive and behavioral impairments that may affect comprehension or expression, as well as those with debilitating physical conditions that could impede study participation, were excluded.

### 2.4. Population

The present study aimed to investigate the multidisciplinary follow-up of patients with neurodegenerative illnesses in two university extension projects. The study enrolled 50 participants, consisting of 14 females aged between 45 and 70 years (mean age ± standard deviation [SD]: 58 ± 8.7 years) and 36 males aged between 38 and 89 years (mean age ± SD: 65 ± 13.2 years), with an overall mean age of 64 years. The majority of participants were retired (70%), and 56% of them were married. Furthermore, 62% of participants identified as having brown skin.

The study population demonstrated significant variability in disease progression and therapy strategy-related traits. Sociodemographic factors, including gender, drug type, and comorbidity profile, were analyzed, and presented in Table 1 and Table 2.

### 2.5. Functional Capacity Tests and Quality of Life Assessment

To evaluate the motor functional capacities of the subjects, two assessments were performed. The first assessment utilized the five times sit to stand test, which is a widely used measure to evaluate the functional capacities of individuals. The second assessment aimed to identify dysfunctional motor control by analyzing the presence of asymmetric activations of symmetrical muscle groups, a phenomenon known as functional dysmetria.

For assessing the quality of life, we utilized the 12-item Short-Form Health Survey, a reliable and validated tool that comprehensively evaluates various aspects of an individual’s quality of life.

#### 2.5.1. Five Times Sit to Stand Test

The five times sit to stand test (FTSST) is a common clinical assessment used to evaluate functional mobility and lower extremity strength in patients with Parkinson’s disease (PD) [20,21]. Parkinson’s disease is a neurodegenerative disorder that affects movement, and evaluating functional mobility is important for assessing disease progression and monitoring treatment efficacy. The FTSST is a standardized test that involves measuring the time it takes for a patient to rise from a seated position to standing and then sitting back down five times consecutively, with the goal of completing the task as quickly as possible.

One of the primary outcomes measured during the FTSST is the time taken to complete the task. In Parkinson patients, this time is often increased compared to healthy individuals, reflecting impaired functional mobility and lower extremity strength. PD patients have slower sit-to-stand performance due to a combination of factors, including bradykinesia, rigidity, and postural instability. These motor symptoms of PD can affect the coordination and timing of movements required for the FTSST, resulting in longer completion times.

The FTSST is a reliable and valid assessment tool for evaluating functional mobility in Parkinson patients, as it provides a standardized and quantifiable measure of lower extremity strength and motor performance [21].

#### 2.5.2. Functional Dysmetria Assessment

Functional dysmetria (FD) is a phenomenon characterized by asymmetric activation of homologous muscle groups on opposite sides of the body and a more general asymmetry in neuromotor control [22]. The term was coined by Rinaldi and Fontani to describe this motor control deficit, which may arise from epigenetic adaptations that results in dysfunction within the neural circuits involved in motor control [22,23].

FD is thought to be a consequence of altered neural plasticity due to various factors, such as genetic, environmental, and developmental influences, resulting in an impaired ability to coordinate movements accurately [22].

Previous studies have investigated the neural mechanisms that underlie functional dysmetria (FD), and they suggest that changes in connectivity and excitability within the cerebellum and cortical motor areas may drive and underpin this phenomenon [23,24,25].

Functional dysmetria (FD) can be evaluated by assessing the activation of the quadriceps muscles during the transition from a supine to sitting position. To measure FD, an operator places their hands symmetrically on the patient’s quadriceps and perceives any asymmetrical activation of these muscle groups during the movement. The measurement of FD is typically given by calculating the difference in alignment between two reference points, which are often the operator’s thumbs, after the patient completes the movement.

#### 2.5.3. Quality of Life Assessment

##### 12-item Short-Form Health Survey

The 12-item Short-Form Health Survey (SF-12) is a widely used tool to assess the quality of life of individuals with various health conditions. It provides a comprehensive evaluation of both physical and mental health by measuring multiple dimensions [21]. The survey comprises 12 questions that are designed to assess various aspects of health, including physical functioning, emotional well-being, and overall health status.

The SF-12 includes eight domains that are developed in response to queries about the limitations and restrictions experienced by individuals in their daily activities. These domains cover a range of topics, including physical activity limitations, restrictions on routine work activities, body pain, self-assessment of health status, including the presence or absence of health problems, and overall perceptions of health.

In addition, the SF-12 also measures lethargy and tiredness, limitations on social interaction brought on by physical or emotional issues, and limitations on conventional role-playing activities as a result of emotional issues, psychological well-being, and distress [16]. These domains provide a comprehensive picture of an individual’s health status and help healthcare professionals to identify areas where interventions may be needed to improve overall quality of life.

Overall, the SF-12 is a valuable tool for assessing quality of life in individuals with various health conditions, including Parkinson’s disease.

#### 2.5.4. REAC Technology

The REAC neurobiological technology and treatments aim to modulate endogenous bioelectric activity at a global level by interacting with the cellular bioelectric activity and optimizing underlying mechanisms, such as neurotransmission.

The REAC technology employs low-intensity radio electric fields, asymmetrically conveyed inside the body to stimulate the cellular bioelectric activity and enhance the communication between neurons. This stimulation can lead to changes in the release of neurotransmitters and neuromodulators, which can have beneficial effects on the nervous system’s overall function. The REAC treatments have been shown to have a positive impact on various neurological and psychiatric conditions, including depression, anxiety, stress-related disorders, and chronic pain.

The device used in this study was BENE 110, produced by ASMED Srl in Florence, Italy.

#### 2.5.5. REAC Neuro Postural Optimization

The REAC NPO treatment was developed to induce an initial and stable electrometabolic and functional reorganization in the brain that may have been altered by dysfunctional adaptive processes, including those with an epigenetic basis [22]. The treatment involves a single administration lasting a few milliseconds that can modify the gradients of endogenous bioelectric activity, leading to a soliton effect that explains the long-term stability of even a single administration [25].

The most notable clinical effect of the REAC NPO treatment is the induction of stable disappearance of functional dysmetria. This effect may be attributed to the treatment’s ability to modulate the bioelectric activity of neurons and enhance communication between brain regions, leading to improved neural plasticity and functional reorganization.

#### 2.5.6. REAC Neuro Psycho Physical Optimization

REAC Neuro Psycho Physical Optimization (NPPO) are a cutting-edge neuromodulation treatment that maximizes human performance by considering the interconnectedness between the nervous system, psychological state, and physical body.

REAC NPPOs treatments have demonstrated efficacy in managing a range of mood and behavioral disorders, including those caused by epigenetic modifications such as in autism spectrum disorder [26,27].

### 2.6. Data Analysis

To assess the normality of the variables before and after the therapy, the Shapiro–Wilk Test was employed. For paired samples with normal distribution, Student’s “T” parametric test was used to compare pre- and post-therapy measurements. In contrast, for variables that did not follow a normal distribution, the Wilcoxon’s non-parametric test was utilized. A significance level of *p* < 0.05 was chosen for both tests.

## 3. Results

### 3.1. Functional Capacity Tests

#### 3.1.1. Functional Dysmetria

**Figure 1:**
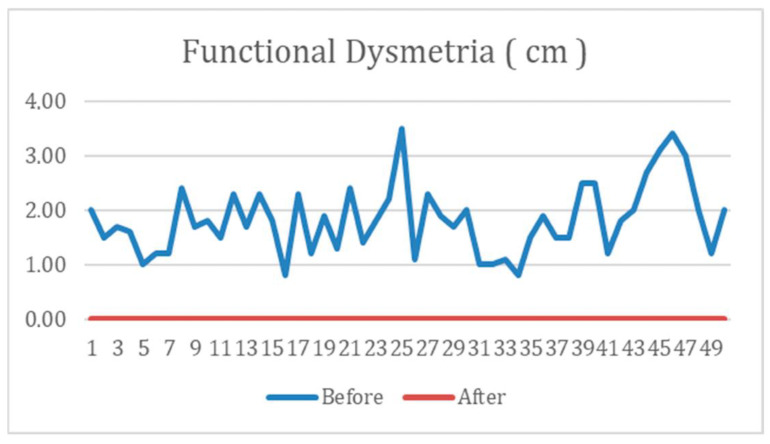
The figure illustrates that, irrespective of the initial values of functional dysmetria (FD) in each subject, administering the REAC-NPO treatment leads to a reset of these values to zero.

The participants in the study underwent an evaluation for functional dysmetria (FD) prior to receiving REAC NPO treatment. The mean FD measurement at baseline was found to be 2 cm. The data were then analyzed using a paired *t*-test to compare the measurements before and after treatment. The analysis revealed a highly significant statistical difference, with a *p*-value of ≤0.001. Notably, following the administration of REAC NPO treatment, the levels of FD in all participants approached zero, as depicted in Figure 1.

#### 3.1.2. Five Times Sit to Stand Test

**Figure 2:**
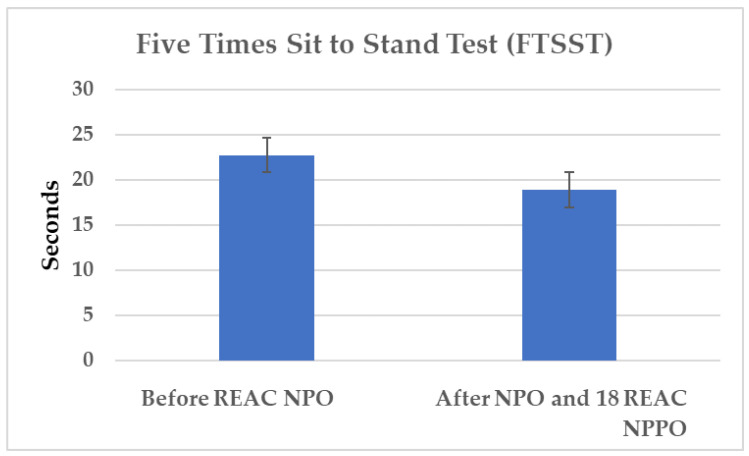
Figure 2 visually depicts the changes in the execution times of the FTSST task before and after the REAC NPO and NPPO treatments. The significant reduction in the median FTSST completion time after the REAC treatments is evident from the comparison of the two sets of bars, indicating an improvement in the functional capacity of individuals with Parkinson’s disease.

The five times sit to stand test (FTSST) was administered both before NPO and after 18 treatment cycles of REAC NPPO to assess the impact of the treatments on the variables pre-FTSST and post-FTSST. The results revealed a significant difference between these variables, as evidenced by a Wilcoxon statistic value (V) of 0.79616 and a corresponding p-value of 0.0003671. Specifically, a significant decrease in the median time to complete the task was observed from FTSST pre 21.98 s, *±* SD 12.18, to FTSST post 17.15 s ± SD 7.26, with a reduction of 4.83 s (Figure 2).

### 3.2. Quality of Life Assessment

#### 12-Item Short-Form Health Survey

To evaluate the effects of REAC NPO and 18 treatment cycles of REAC NPPO on the physical and mental health of participants, the 12-item Short-Form Health Survey (SF-12) questionnaire was administered both before and after the intervention. The SF-12 is a widely used survey tool that measures both physical and mental health components.

**Figure 3:**
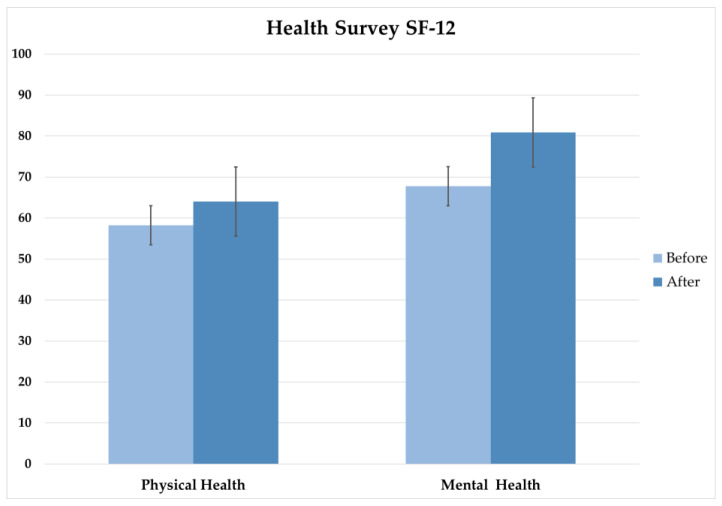
The 12-item Short-Form Health Survey, physical and mental health cluster results pre- and post-intervention.

Statistical analyses were performed to evaluate changes in physical and mental health clusters between pre- and post-intervention. Specifically, a Wilcoxon signed-rank test was used to compare pre- and post-intervention scores for the physical health cluster. The resulting *p*-value of ≤0.014757 indicates a significant improvement in physical health following the REAC protocols (Figure 3).

For the mental health cluster, a paired t-test was used to compare pre- and post-intervention scores. The resulting *p*-value of ≤0.001 indicates a significant improvement in mental health following the REAC protocols (Figure 3).

## 4. Discussion

The aim of this study was to evaluate the impact of REAC NPO and a single cycle of REAC NPPO 18 treatment on the physical and mental well-being of individuals diagnosed with Parkinson’s disease. Specifically, we aimed to improve their functional capacity and quality of life through REAC neuromodulation treatments targeting mood and behavioral disorders.

The results regarding functional capabilities are generally intriguing and affirmative, warranting a detailed discussion to comprehend the underlying reasons for the observed outcomes.

Functional dysmetria is a complex maladaptive condition that affects neurological, psychological, and physical functioning and is likely influenced by epigenetic factors, as evidenced by changes in fluctuating asymmetry of morphological asymmetries. REACNPO treatment offers a promising approach to address this condition by promoting a more efficient and functional encephalic electro-metabolic reorganization [25] that can optimize the body’s response to epigenetic decay.

In this study, we have shown that NPO can effectively reduce functional dysmetria in individuals with Parkinson’s disease, highlighting the dysfunctional component of the condition that can be addressed through this treatment approach.

Moreover, the results of the study suggest that REAC NPO and NPPOs treatments have a positive effect on the functional capacity of individuals with Parkinson’s disease, as demonstrated by the significant improvement in their ability to complete the FTSST. Specifically, the median time to complete the FTSST was significantly reduced after the treatments, which suggests that the REAC treatments may have improved in individuals with Parkinson’s disease strength, balance, and overall physical function.

The findings are consistent with previous studies that have investigated the effects of REAC treatments on physical function in older adults [28].

The results relating to the quality-of-life assessment obtained using the 12-item Short-Form Health Survey (SF-12) questionnaire was used to measure physical and mental health components before and after the intervention.

The statistical analysis showed significant improvements in both physical and mental health following the REAC NPO and NPPOs treatments. The Wilcoxon signed-rank test showed a significant improvement in physical health, while the paired t-test showed a significant improvement in mental health. These findings are consistent with previous studies that have shown the positive effects of REAC on physical and mental health.

The implications of these SF-12 results are significant, as the findings suggest that the REAC NPO and NPPOs treatment protocols can be an effective intervention results for improving both physical and mental health. This is particularly relevant in the context of the post-COVID-19 pandemic effects, where mental health issues have become increasingly prevalent.

The findings of this study have important implications, suggesting that REAC NPO and NPPOs neuromodulation treatments may be an effective intervention for improving physical function in older adults and individuals with Parkinson’s disease. This is particularly relevant given the increasing prevalence of age-related conditions that can lead to functional decline.

In addition to improving specific ailments, the beneficial impact of REAC NPO and NPPOs treatments on physical function can extend to enhancing overall health and wellbeing in older adults. This improvement can help them maintain their independence and quality of life. By aiding in the recovery and preservation of physical function, REAC treatments can contribute to a better quality of life, enabling older adults and individuals with Parkinson’s disease to engage in daily activities and maintain their sense of autonomy. This highlights the potential of REAC NPO and NPPOs treatments approach to promoting healthy aging and addressing the challenges faced by older adults also with Parkinson’s disease.

## 5. Conclusions

PD is a complex disorder with both motor and non-motor symptoms that can significantly impact daily functioning. Understanding the correlations between motor and non-motor symptoms is important for the effective management of the condition, and the use of advanced technologies, such as REAC NPO and NPPO neuromodulation treatments may offer new avenues for improving neuropsychic and physical performance in PD patients. More research is needed to explore the effects of multiple courses of REAC NPPOs treatments in individuals with Parkinson’s disease to optimize treatment protocols for maximum benefit.

## Figures and Tables

**Table 1:** Distribution of the socioeconomic variables of individuals, according to gender.

Variables	Categories	Female	*N* = 14%	Male	*N* = 36%
Age	30–49 years	1	7.1%	4	11.1%
	50–69 years	12	85.8%	20	55.5%
	Over 70 years	1	7.1%	12	33.3%
Color/Race	White	3	21.4%	8	22.2%
	Brown	9	64.2%	22	61.1%
	Black	2	14.2%	3	8.3%
	Indigenous	0	0%	2	5.5%
	Yellow	0	0%	1	2.7%
Marital Status	Single	1	7.1%	7	19.4%
	Stable union	1	7.1%	2	5.5%
	Married	5	35.7%	21	58.3%
	Divorced	5	35.7%	6	16.6%
	Widower	2	14.2%	0	0%
Comorbidity	Yes	12	85.8%	21	58.3%
	No	2	14.2%	15	41.7%
Continuous medication use	Yes	14	100%	36	100%
	No	0	0%	0	0%

**Table 2:** Overview of medications and associated disease/comorbidity.

Medications	Patients: *N* = 50		Disease/Comorbidity	Patients *N* = 50	
	Amount	%		Amount	%
Levodopa	47	94%	Parkinson	50	100%
Benzerazide hydrochloride	39	78%	Arterial Hypertension	21	42%
Pramipexole dihydrochloride	21	42%	Anxiety	17	34%
Losartan	9	18%	Depression	6	12%
Biperiden hydrochloride	6	12%	Diabetes	5	10%
Metformin	5	10%	Dyslipidemia	2	4%
Acetylsalicylic acid	4	8%	Cancer	2	4%
Amantadine hydrochloride	4	8%	Osteoporosis	1	2%
Amlodipine Bensylate	4	8%	Arthritis	1	2%
Biperiden	3	6%	Fibromyalgia	1	2%
Melatonin	3	6%	Diverticulitis	1	2%
Amitriptyline hydrochloride	2	4%	Urinary Incontinence	1	2%
Sinvastatin	2	4%	Asthma	1	2%
Fluoxetine hydrochloride	2	4%	Hypothyroidism	1	2%
Verapamil hydrochloride	2	4%	Rheumatism	1	2%
Clozapine	1	2%	Cataract	1	2%
Domperidone	1	2%	Coronary Artery Disease	1	2%
Clonazepam	1	2%			
Paroxetine hydrochloride	1	2%			
Metroprolol succinate	1	2%			
Ramipril	1	2%			
Carbomazepine	1	2%			
Alprazolam	1	2%			
Hydrochlorothiazide	1	2%			
Rosuvastatin calcium	1	2%			
Enalapril maleate	1	2%			
Valsatarn	1	2%			
Oxalate of escitalopram	1	2%			
Empagliflozin	1	2%			
Levothyroxine sodium	1	2%			

## Data Availability

All study data are present in the manuscript.

## References

[1] Barreto K.S., Oliveira J., Reis L.D., Ribeiro T.G., Kauark R.B.G. (2022). Non-motor symptoms fluctuations in patients with Parkinson’s disease at the Clinical Hospital of Salvador, Bahia. Dement. Neuropsychol..

[2] Kumar A., Patil S., Singh V.K., Pathak A., Chaurasia R.N., Mishra V.N., Joshi D. (2022). Assessment of Non-Motor Symptoms of Parkinson’s Disease and Their Impact on the Quality of Life: An Observatiobnal Study. Ann. Indian Acad. Neurol..

[3] Cao Y., Si Q., Tong R., Zhang X., Li C., Mao S. (2023). Abnormal dynamic functional connectivity changes correlated with non-motor symptoms of Parkinson’s disease. Front. Neurosci..

[4] Kwon K.Y., Park S., Kim R.O., Lee E.J., Lee M. (2022). Associations of cognitive dysfunction with motor and non-motor symptoms in patients with de novo Parkinson’s disease. Sci. Rep..

[5] Mantovani E., Zucchella C., Argyriou A.A., Tamburin S. (2023). Treatment for cognitive and neuropsychiatric non-motor symptoms in Parkinson’s disease: Current evidence and future perspectives. Expert. Rev. Neurother..

[6] Schaffner S.L., Kobor M.S. (2022). DNA methylation as a mediator of genetic and environmental influences on Parkinson’s disease susceptibility: Impacts of alpha-Synuclein, physical activity, and pesticide exposure on the epigenome. Front. Genet..

[7] Polito L., Greco A., Seripa D. (2016). Genetic Profile, Environmental Exposure, and Their Interaction in Parkinson’s Disease. Parkinsons Dis..

[8] Talavera Andujar B., Aurich D., Aho V.T.E., Singh R.R., Cheng T., Zaslavsky L., Bolton E.E., Mollenhauer B., Wilmes P., Schymanski E.L. (2022). Studying the Parkinson’s disease metabolome and exposome in biological samples through different analytical and cheminformatics approaches: A pilot study. Anal. Bioanal. Chem..

[9] Angelopoulou E., Paudel Y.N., Papageorgiou S.G., Piperi C. (2022). Environmental Impact on the Epigenetic Mechanisms Underlying Parkinson’s Disease Pathogenesis: A Narrative Review. Brain Sci..

[10] Khedr E.M., Abdelrahman A.A., Elserogy Y., Zaki A.F., Gamea A. (2020). Depression and anxiety among patients with Parkinson’s disease: Frequency, risk factors, and impact on quality of life. Egypt. J. Neurol. Psychiatry Neurosurg..

[11] Marsh L. (2013). Depression and Parkinson’s disease: Current knowledge. Curr. Neurol. Neurosci. Rep..

[12] Papapetropoulos S., Ellul J., Argyriou A.A., Chroni E., Lekka N.P. (2006). The effect of depression on motor function and disease severity of Parkinson’s disease. Clin. Neurol. Neurosurg..

[13] Prange S., Klinger H., Laurencin C., Danaila T., Thobois S. (2022). Depression in Patients with Parkinson’s Disease: Current Understanding of its Neurobiology and Implications for Treatment. Drugs Aging.

[14] Svensson E., Farkas D.K., Gradus J.L., Lash T.L., Sorensen H.T. (2016). Adjustment disorder and risk of Parkinson’s disease. Eur. J. Neurol..

[15] Barer Y., Chodick G., Glaser Chodick N., Gurevich T. (2022). Risk of Parkinson Disease Among Adults With vs Without Posttraumatic Stress Disorder. JAMA Netw. Open.

[16] Morgan M.A., Kelber M.S., Bellanti D.M., Beech E.H., Boyd C., Galloway L., Ojha S., Garvey Wilson A.L., Otto J., Belsher B.E. (2022). Outcomes and prognosis of adjustment disorder in adults: A systematic review. J. Psychiatr. Res..

[17] Levin M., Pezzulo G., Finkelstein J.M. (2017). Endogenous Bioelectric Signaling Networks: Exploiting Voltage Gradients for Control of Growth and Form. Annu. Rev. Biomed. Eng..

[18] Faul F., Erdfelder E., Lang A.G., Buchner A. (2007). G*Power 3: A flexible statistical power analysis program for the social, behavioral, and biomedical sciences. Behav. Res. Methods.

[19] Kang H. (2021). Sample size determination and power analysis using the G*Power software. J. Educ. Eval. Health Prof..

[20] Munoz-Bermejo L., Adsuar J.C., Mendoza-Munoz M., Barrios-Fernandez S., Garcia-Gordillo M.A., Perez-Gomez J., Carlos-Vivas J. (2021). Test-Retest Reliability of Five Times Sit to Stand Test (FTSST) in Adults: A Systematic Review and Meta-Analysis. Biology.

[21] Duncan R.P., Leddy A.L., Earhart G.M. (2011). Five times sit-to-stand test performance in Parkinson’s disease. Arch. Phys. Med. Rehabil..

[22] Fontani V., Rinaldi A., Rinaldi C., Araldi L., Azzara A., Carta A.M., Casale N., Castagna A., Del Medico M., Di Stasio M. (2022). Long-Lasting Efficacy of Radio Electric Asymmetric Conveyer Neuromodulation Treatment on Functional Dysmetria, an Adaptive Motor Behavior. Cureus.

[23] Mura M., Castagna A., Fontani V., Rinaldi S. (2012). Preliminary pilot fMRI study of neuropostural optimization with a noninvasive asymmetric radioelectric brain stimulation protocol in functional dysmetria. Neuropsychiatr. Dis. Treat..

[24] Rinaldi S., Fontani V., Castagna A. (2011). Brain activity modification produced by a single radioelectric asymmetric brain stimulation pulse: A new tool for neuropsychiatric treatments. Preliminary fMRI study. Neuropsychiatr. Dis. Treat..

[25] Rinaldi S., Mura M., Castagna A., Fontani V. (2014). Long-lasting changes in brain activation induced by a single REAC technology pulse in Wi-Fi bands. Randomized double-blind fMRI qualitative study. Sci. Rep..

[26] Rinaldi A., Maioli M., Marins Martins M.C., de Castro P.C.F., de Oliveira Silva N.A.P., de Mattos J.A.V., Fontani V., Rinaldi S. (2021). REAC Non-invasive Neurobiological Stimulation for Mitigating the Impact of Internalizing Disorders in Autism Spectrum Disorder. Adv. Neurodev. Disord..

[27] Rinaldi A., Martins M.C.M., Maioli M., Rinaldi S., Fontani V. (2023). REAC Noninvasive Neurobiological Stimulation in Autism Spectrum Disorder for Alleviating Stress Impact. Adv. Neurodev. Disord..

[28] Fontani V., Rinaldi S., Castagna A., Margotti M.L. (2012). Noninvasive radioelectric asymmetric conveyor brain stimulation treatment improves balance in individuals over 65 suffering from neurological diseases: Pilot study. Ther. Clin. Risk Manag..

